# Province- and Individual-Level Influential Factors of Depression: Multilevel Cross-Provinces Comparison in China

**DOI:** 10.3389/fpubh.2022.893280

**Published:** 2022-05-06

**Authors:** Yue Gou, Nianwei Wu, Jing Xia, Yanjun Liu, Huawu Yang, Haibo Wang, Tong Yan, Dan Luo

**Affiliations:** ^1^Department of General Surgery, The Center of Gastrointestinal and Minimally Invasive Surgery, The Third People's Hospital Chengdu, The Affiliated Hospital of Southwest Jiaotong University, Chengdu, China; ^2^Department of General Surgery, Center for Obesity and Metabolic Health, The Third People's Hospital of Chengdu, The Affiliated Hospital of Southwest Jiaotong University, Chengdu, China; ^3^Research Center for Obesity and Metabolic Health, College of Medicine, Southwest Jiaotong University, Chengdu, China; ^4^Medical Research Center, The Third People's Hospital of Chengdu, The Affiliated Hospital of Southwest Jiaotong University, Chengdu, China; ^5^Department of Health and Social Behavior, West China School of Public Health, West China Fourth Hospital, Sichuan University, Chengdu, China

**Keywords:** Chinese, depression, multilevel analysis, province-level, individual-level

## Abstract

Rapid social change has given rise to a general increase in psychological pressure, which has led to more and more Chinese people suffering from depression over the past 30 years. Depression was influenced not only by individual factors but also by social factors, such as economy, culture, politics, etc. These social factors were measured at the national, provincial, or community levels. However, little literature reported the influence of province-level factors on the depression of Chinese. This study examined the effects of province-level and individual-level factors on depression of Chinese respondents aged 16–97 years. We conducted a multilevel analysis of the 2018 wave survey of the Chinese Family Panel Studies (CFPS), with 19,072 respondents nested within the 25 Chinese provinces. Data for the province-level were extracted from the National Bureau of Statistics of China, including three predictors: gross regional product (GRP) per capita, expenditure for social security and employment (ESSE), and rural and urban household income inequality. Depression was measured with the eight-item short version of the Center for Epidemiologic Studies Depression Scale (CES-D8). The study found that respondents who were female, 30–59 years, divorced or widowed, less educated, rural residents, less body mass index (BMI), or had lower household income tended to report higher levels of depressive symptoms. After adjustment for individual-level features, a significant effect of provinces still survived. The respondents who lived in a province with higher GRP, higher ESSE, or smaller rural and urban household income inequality reported lower depressive symptoms. Our results demonstrated that individual features did not fully explain depression. Economic and social factors appeared to impact depression and have to be considered when the government planned for improved public depression. Meanwhile, our research also provided a suggestion for the government of some provinces to investigate and improve depression.

## Introduction

Depression is characterized by sadness, loss of interest or pleasure, feelings of guilt or low self-worth, disturbed sleep or appetite, feelings of tiredness, and poor concentration (1). Depression can be long-lasting or recurrent, substantially impairing an individual's quality of life, the function of daily activities, and even suicide if left untreated (2–6). At a global level, more than 264 million people were affected by depression in 2017. In addition, depression is one of the three chronic non-communicable diseases (NCDs, including low back pain, headache disorders, and depressive disorders), which have prevailed as three of the top four leading causes of years lived with disability (YLDs). They collectively caused 162 million YLDs in 2017, which accounted for nearly one-quintile of YLDs globally (7).

The economy has developed at an unprecedented rate in China over the past 30 years. Rapid social change gives rise to a general increase in psychological pressure, which leads to more and more Chinese people suffering from depression (8). Yang et al. researched rapid health transition in China from 1990 to 2010, and found that depression was one of the leading causes of disability-adjusted life years (DALYs) in 2010. Additionally, major depression was the second leading cause of disability (9). According to the World Health Organization, more than 54 million Chinese people suffered from depression in 2015, representing 4.2% of the population (1). A case-control study with 1,055 participants randomly selected from 23 geographically representative sites in China demonstrated that high depression symptom is the most critical risk factor of suicide (10). These studies showed that the high prevalence of depression and its severe consequences had been an intense societal challenge in China.

Given the prevalence of depression, serious consequences caused by depression, and the burden of depression rising globally (11, 12), a World Health Assembly resolution passed in May 2013 has appealed to a comprehensive, coordinated response to mental disorders at the country level (5). Many scholars and institutions are increasingly interested in depression and do a lot of research on depression from various perspectives. Generally, most studies all demonstrated that basic sociodemographic factors are associated with depression, such as gender, age, marital status, educational attainment, occupation so on (13–16). For example, a multilevel cross-national study suggested that females were at higher risk of depression than males; higher education had a lower level of depression (17). In addition, social factors and the natural environment all have significant effects on depression. The results from three studies suggest that long-term exposure to air pollution may increase the risk of depression (18–20). In recent decades, more researchers have been concerned about the impact of social circumstances on depression. Some scholars are finding that the economic level of the country or district, income inequality, community building, and other social circumstances have a significant influence on depression despite adjusting the basic demographic characteristics (21–25). A meta-analysis showed that depression has a high prevalence in low socioeconomic status (26). A population-based, representative longitudinal study found that women living in higher-income inequality were associated with a higher risk of depression (23). Those studies together suggested that it is helpful to reduce the prevalence of depression when the government makes the related macro policies with taking the macro influence factors of depression into account. So far, many studies have already shown that depression is influenced by complex interactions between cultural, psychological, and biological factors (27–30). Many studies about depression in China have been conducted from individual-level or community-level (31, 32). However, few studies pay attention to relevant factors at the province level.

For filling the gap mentioned above, we developed this study. We combined the individual-level characteristics and province-level factors to investigate factors influencing depression. Data for individual-level characteristics were derived from the China Family Panel Studies (CFPS); thus, data province-level factors were extracted from the National Bureau of Statistics of China. This study will confirm the sociodemographic and province-level factors associated with depression and provide a reference for the government to make macro policies.

## Materials and Methods

### Sources of Data and Study Population

The data set was derived from the CFPS. The CFPS, conducted biennially by the Institute of Social Science Survey (ISSS) at Peking University, is a nearly nationwide, comprehensive, longitudinal social survey intended to serve research needs on a large variety of social phenomena in contemporary China (33). The CFPS baseline survey was launched in 2010 whose sample covers 25 provinces/municipalities/autonomous regions (excluding Hong Kong, Macao, Taiwan, Xinjiang, Tibet, Qinghai, Inner Mongolia, Ningxia, and Hainan), representing 95% of the Chinese population. The 2010 baseline survey interviewed 14,960 households and 42,590 individuals, and it is China's first large-scale academically oriented longitudinal survey project. The CFPS adopted a multi-stage stratification method to generate a nationally representative sample and carried out a three-stage sampling process. In the first and second stages, one hundred sixty-two county-level units and 640 villages/communities were randomly selected. Fourteen thousand nine hundred sixty households were established in the third stage according to the “Probability-Proportional-to-Size” (PPS) sampling strategy. The CFPS gathers a wealth of information covering economic activities, education outcomes, family dynamics and relationships, migration, and health (33–35).

This study utilized data from the most recent China Family Panel Studies (CFPS) wave in 2018. Although CFPS official has not yet published the 2018 wave of CFPS sampling weights, we can still select a nationally representative sample based on the official guide (36). Finally, we chose the national resampling sample of 2018 CFPS as our study population, including 21,579 observations. We restricted the study population to those aged ≥16 years because they had completed their questionnaires. After excluding participants with missing information on demographic and depression scale variables, we got 19,072 participants for the analysis.

### Outcome Variable: Depression

The 2018 CFPS questionnaire employed the eight-item short version of the Center for Epidemiologic Studies Depression Scale (CES-D8), one of the most widely used self-evaluation scales originally developed by Radloff (37, 38). In 2018, CFPS questionnaire, the items that composed the scale were the following: (N1) “I am in a low spirit.,” (N2) “I find it difficult to do anything,” (N3) “I cannot sleep well,” (N4) “I feel happy,”. (N5) “I feel lonely.” (N6) “I have a happy life.” (N7) “I feel sad.” (N8) “I feel that I cannot continue with my life.” Then respondents were asked to indicate how often in the week before the survey they felt or behaved above mentioned. Responses to items on the CES-D are specified using a 4-point Likert scale where 0 represents the category “Never (<1 day/week),” 1 “Sometimes (1–2 days/week),” 2 “Often (3–4 days/week),” 3 “Most of the time (5–7 days/week).” Two positive worded items (N4 and N6) were reverse-coded. Eventually, a total possible score ranges from 0 to 24, with higher scores indicating a greater frequency and severity of depressive symptoms. The reliability and validity of the CES-D8 were confirmed across gender and countries (21, 39). The internal consistency reliability coefficients of this study's 8-item scale were satisfactory (Cronbach α = 0.77).

### Individual-Level Measures

We included the following individual demographic variables: gender (female, male); age consisted of five groups (16–29 years, 30–44 years, 45–59 years, 60–74 years, and older than 74 years); educational attainment was a categorical variable (no formal education, primary school, junior high, senior high and college or higher); marital status (married and living with a spouse, unmarried and divorced or widowed); and per capita household net income in RMB (yuan) per years (measured in quartiles). Moreover, due to China's special dual household registration system (Hukou) (40), we also consider including a type of residence to investigate rural and urban respondent's depression. This study used the definition of urban–rural classification based on the National Bureau of Statistics of China. We included the objective health indicator body mass index (BMI) from self-reported height and body weight and treated it as four dichotomous groups (below 18.5, 18.5–23.9, 24–27.9, and ≥28.0).

### Province-Level Measures

Previous studies have shown that depression might be impacted by economic level, income inequality, social wellbeing, and so on (21, 23). So, at the province level, there were three predictors included in this study: (1) gross region product (GRP) per capita, as a measure of the overall size of the economy for every province; (2) the ratio of per capita disposable income of urban and rural (RUR), roughly measuring the degree of rural and urban household income inequality; and (3) expenditure for social security and employment (ESSE), as a predictor of the people's wellbeing. They were all viewed as continual variables. These province-level data were extracted from the National Bureau of Statistics of China (detail information on the official website: http://www.stats.gov.cn/tjsj/ndsj/).

### Statistical Analysis

This study viewed the outcome variable, CES-D8, as a continuous variable, with a possible value from 0 to 24. We were considering the prominent hierarchical characteristics of the data, individuals (level-1 units) clustered within provinces (level-2 units), meaning that the standard assumption of independent observations is likely to be violated. A two-level multilevel linear regression model was used to assess the effects of individual sociodemographic characteristics and contextual levels on depression. We can correct the biases in parameter estimates for the clustering data using multilevel analysis, thus getting more accurate parameter estimates (41). The multilevel model can be described as follows with the restricted maximum likelihood method (REML) using the command “mixed” in Stata15.0 (Stata Corp LLC, College Station, Texas, US) (42):


$$\begin{array}{r} Y_{ij}=\;\beta_{00}+\beta_{10}X_{ij}+\beta_{01}Z_{j}+\mu_{0j}+e_{ij} \end{array}$$

where *i*: individual subscript; *j*: province subscript; *Y*: the value of CES-D8; *X*: a vector of individual-level variables, including gender, age, education, marital status, per capita household net income, residence (urban/rural), BMI; *Z*: a vector of province-level variables, including GRP, RUR, ESSE; μ_0*j*_: residual error at the province level; *e*_*ij*_: residual error at the individual level. This equation is a comparatively straightforward model, but we have assumed that μ_0*j*_ and *e*_*ij*_ have zero expectations (41, 42).

First, a null model with only a constant term in the fixed part was fit, which allowed us to detect the possible existence of contextual variation in depression across provinces. After that, we included the individual-level variables in the model. Then three province-level variables were added to the model, respectively. Finally, all variables, including individual-level and province-level, were added to the model.

In addition, a robustness check was performed. We treated the outcome variable as a binary variable and used the multilevel logistic regression to fit the model again, following the same modeling strategy.

Finally, it must be cautious that our statistical analyses do not offer a causal explanation, so when the term “explain” is used in the following sections, it should be understood in a statistical rather than a causal relationship sense (39). *p* < 0.05 was defined as statistical significance.

## Results

### Descriptive Statistic of the Sample

As shown in Table 1, the overall depression scores among these 19,072 respondents were relatively low (mean = 5.53). Of all respondents, 9,589 (50.28%) were men, and 9,483 (49.72%) were women; the age ranged from 16 to 97 years old, with 45–59 years old (29.87%) respondents being the largest number, followed by group 30–44 years old (24.75%). The majority of them were married or were living with their spouses. There were 5,790 (30.36%) respondents having junior high educational attainment. 9,125 (47.85%) and 9,947 (52.15%) respondents respectively came from rural and urban. BMI for most (54.17%) respondents was normal, between 18.5 and 23.9.

**Table 1:** Basic characteristics for the individual- and province-level variables.

**Variables**	**N**	**Percentage or mean (SD)**
**Outcome**
Depression scores from CES-D8	19,072	5.53 (3.95)
**Individual-level characteristics**
**Gender**
Female	9,589	50.28%
Male	9,483	49.72%
**Age Group**
16–29	3,801	19.93%
30–44	4,720	24.75%
45–59	5,697	29.87%
60–74	4,088	21.43%
Above 74	7,66	4.02%
**Marital status**
Married & living with spouse	14,815	77.68%
Unmarried	3,054	16.01%
Divorced or widowed	1,203	6.31%
**Education Attainment**
No formal education	4,108	21.54%
Primary school	3,847	20.17%
Junior high	5,790	30.36%
Senior high	4,332	22.71%
College or higher	995	5.22%
**Rural/Urban residence**
Rural	9,125	47.85%
Urban	9,947	52.15%
**BMI**
Below 18.5	1,623	8.51%
18.5–23.9	10,331	54.17%
24–27.9	5,475	28.71%
28 and above	1,643	8.61%
**Per capita household net income**
Quartile 1(lowest)	4,672	24.50%
Quartile 2	5,485	28.76%
Quartile 3	5,131	26.90%
Quartile 4(highest)	3,784	19.84%
**Province-level variables**
GRP per capita (thousand)	25	68.32 (31.53)
RUR	25	2.50 (0.36)
ESSE (billion)	25	95.27 (33.17)

*BMI, body mass index; CES-D8, the eight-item short version of the center for epidemiologic studies depression scale; ESSE, expenditure for social security and employment; GRP, gross regional product; RUR, the ratio of per capita disposable income of urban and rural*.

At the province level, this study included 25 provinces of China-related data. Averagely, the GRP per capita of 25 provinces was 68,300 yuan; per capita disposable income of urban residents was 2.50 times that of rural residents; the expenditure for social security and employment for 25 provinces was about 95.27 billion.

### The Null Model

Model 1 in Table 2 showed the null model (just with a constant term in the fixed part, while without any predictors at any level), which indicated statistically significant variations in the severity of depressive symptoms at both the individual and the province level. The size of the intraclass correlation (ICC) in Model 1 was 0.026 (*p* < 0.01), suggesting that 2.6% of the variance in the dependent variable can be attributed to the province level. Though the ICC seemed to be so small, related studies have already shown that we cannot ignore it when explaining province-level measures' effects (43–45). Hence it is rational for us to conduct multilevel statistics.

**Table 2:** Results for the two-level multilevel linear regression models.

**Variables**	**Model 1**	**Model 2**	**Model 3**	**Model 4**	**Model 5**	**Model 6**
**Individual-level variable**
**Gender (ref: female)**
Male		−0.623^***^ (0.057)	-0.623^***^ (0.057)	−0.623^***^ (0.057)	-0.623^***^ (0.057)	−0.624^***^ (0.057)
**Age group (ref: 16–29)**
30–44		0.432^***^ (0.108)	0.434^***^ (0.108)	0.432^***^ (0.108)	0.432^***^ (0.108)	0.434^***^ (0.108)
45–59		0.344^**^ (0.113)	0.347^**^ (0.113)	0.343^**^ (0.113)	0.346^**^ (0.113)	0.350^**^ (0.113)
60–74		−0.062 (0.122)	-0.055 (0.122)	−0.059 (0.122)	-0.060 (0.122)	−0.050 (0.122)
Above 74		−0.131 (0.182)	-0.125 (0.182)	−0.127 (0.182)	-0.126 (0.182)	−0.114 (0.182)
**Marital status (ref: married and living with spouse)**
Unmarried		0.043 (0.111)	0.041 (0.111)	0.043 (0.111)	0.045 (0.111)	0.045 (0.111)
Divorced or widowed		1.378^***^ (0.120)	1.378^***^ (0.120)	1.377^***^ (0.120)	1.380^***^ (0.120)	1.379^***^ (0.120)
**Educational attainment (ref: no formal education)**
Primary school		−0.693^***^ (0.090)	-0.691^***^ (0.090)	−0.693^***^ (0.090)	-0.692^***^ (0.090)	−0.689^***^ (0.090)
Junior high		−0.869^***^ (0.090)	-0.866^***^ (0.090)	−0.868^***^ (0.090)	-0.869^***^ (0.090)	−0.864^***^ (0.090)
Senior high		−1.185^***^ (0.103)	-1.181^***^ (0.102)	−1.184^***^ (0.103)	-1.182^***^ (0.102)	−1.177^***^ (0.102)
College or higher		−1.141^***^ (0.157)	-1.134^***^ (0.157)	−1.137^***^ (0.157)	-1.143^***^ (0.157)	−1.133^***^ (0.157)
**Rural/Urban residence (ref: rural)**	
Urban		−0.251^***^ (0.061)	-0.247^***^ (0.061)	−0.248^***^ (0.061)	-0.247^***^ (0.061)	−0.238^***^ (0.061)
**BMI (ref: 18.5-23.9)**	
Below 18.5		0.561^***^ (0.104)	0.561^***^ (0.104)	0.561^***^ (0.104)	0.562^***^ (0.104)	0.561^***^ (0.104)
24–27.9		−0.279^***^ (0.065)	-0.279^***^ (0.065)	−0.279^***^ (0.065)	-0.279^***^ (0.065)	−0.279^***^ (0.065)
28 and above		−0.328^**^ (0.102)	-0.328^**^ (0.102)	−0.327^**^ (0.102)	-0.329^**^ (0.102)	−0.328^**^ (0.102)
**Per capita household net income (ref: quartile 1)**	
Quartile 2		−0.554^***^ (0.078)	-0.550^***^ (0.078)	−0.553^***^ (0.078)	-0.557^***^ (0.078)	−0.552^***^ (0.078)
Quartile 3		−0.868^***^ (0.083)	-0.862^***^ (0.083)	−0.864^***^ (0.083)	-0.872^***^ (0.083)	−0.861^***^ (0.083)
Quartile 4		−0.840^***^ (0.097)	-0.831^***^ (0.097)	−0.825^***^ (0.097)	-0.848^***^ (0.096)	−0.828^***^ (0.097)
**Province-level variable**
RUR			0.893^***^ (0.229)			0.610^**^ (0.215)
GDP				−0.065^*^ (0.033)		−0.024 (0.026)
ESSE					-0.010^***^ (0.002)	−0.008^***^ (0.002)
Constant	5.529^***^ (0.133)	7.670^***^ (0.186)	5.413^***^ (0.608)	8.097^***^ (0.281)	8.611^***^ (0.288)	7.044^***^ (0.733)
*N*	19,072	19,072	19,072	19,072	19,072	19,072

*BMI, body mass index; ESSE, expenditure for social security and employment; GRP, gross regional product; RUR, the ratio of per capita disposable income of urban and rural. β and standard errors (standard errors in parentheses)*;

* *p < 0.05*,

** *p < 0.01*,

*** *p < 0.001*.

### Individual-Level Characteristics and Depression

Association between depression and individual-level characteristics is mainly reflected in fixed effects interpreted as the average effect of the variable across the province (25). Model 2 added all individual-level variables, including gender, age, marital status, education attainment, rural/urban residence, BMI, and household net income. The results demonstrated that male respondents (β = −0.623, *p* < 0.001), urban respondents (β = −0.251, *p* < 0.001), high-income household (β = −0.840, *p* < 0.001) were more likely to report lower depression scores suggesting a slight frequency and severity of depressive symptoms. We find that significant differences emerge between married/living with spouse and divorced/widowed participant's depressive scores (*p* < 0.001), but there are no significant differences between married/living with spouse and unmarried. Respondents aged 30–44 years (β = 0.432, *p* < 0.001), 45-59 (β = 0.344, *p* < 0.01) years reported higher depression scores. Furthermore, our study showed that higher educational attainment and values of BMI were at a low risk of depression (*p* < 0.001).

### Province-Level Variables and Depression

After controlling the individual-level variables, we added the variables of province-level (RUR, GRP per capita, ESSE) into models 3–5, respectively. We find that those respondents living in provinces with higher economic level (β = −0.065, *p* < 0.05) and better people's wellbeing (β = −0.010, *p* < 0.001) were more likely to report low depression scores. The results also suggested that respondents living in provinces with higher RUR were more likely to report higher depression scores (β = 0.893, *p* < 0.001). In the end, all variables, including individual-level and province-level variables, were added to model 6. As showed by model 6, all individual characteristics were still significantly associated with the respondent's depression scores. Still, the effect of GRP per capita became statistically insignificant, suggesting that the GRP per capita in depression scores among these respondents was somewhat confounded by the individual-level variables and other two province-level variables of RUR (β = 0.610, *p* < 0.01) and ESSE (β = −008, *p* < 0.001).

### Residual Analysis

**Figure 1:**
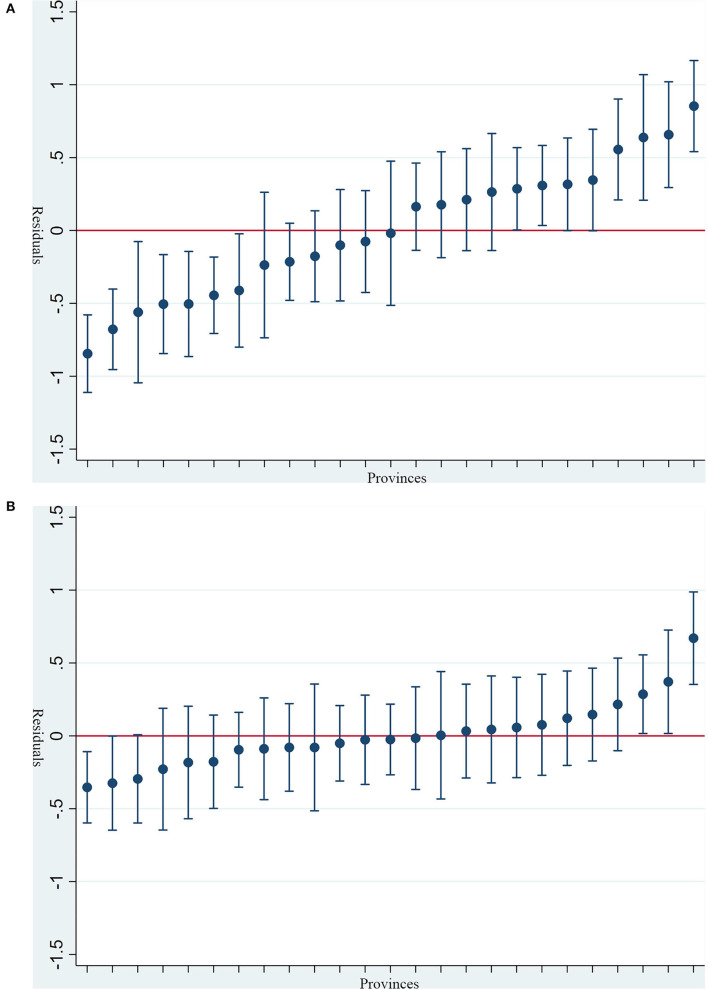
**(A)** Caterpillar plot of provinces residual and ~95% CIs vs. ranking (after controlling the individual-level characteristics; sequencing from left to right: Sichuan, Shandong, Shanghai, Zhejiang, Jiangsu, Henan, Liaoning, Tianjin, Hebei, Heilongjiang, Hubei, Anhui, Beijing, Yunnan, Jilin, Guangxi, Fujian, Shanxi, Guangdong, Hunan, Jiangxi, Shaanxi, Chongqing, Gansu, Guizhou). **(B)** Caterpillar plot of provinces residual and ~95% CIs vs. ranking (after controlling the individual-level characteristics and three province-level variables; sequencing from left to right: Shandong, Sichuan, Yunnan, Shanghai, Gansu, Zhejiang, Henan, Liaoning, Anhui, Tianjin, Shanxi, Guangxi, Hebei, Jiangsu, Beijing, Heilongjiang, Fujian, Guizhou, Jilin, Hubei, Shaanxi, Jiangxi, Hunan, Chongqing, Guangdong).

Random-intercept predictions μ_0*j*_ are sometimes viewed as measures of institutional performance (42)—in this study, how much depression scores the provinces add for individuals. Therefore, the caterpillar plot was depicted to demonstrate the situation of depression in 25 provinces of China. Figure 1A shows the caterpillar plot after controlling the individual-level characteristics. The results indicated that respondents living in Guangdong, Shaanxi, Chongqing, Gansu, and Guizhou were more likely to report higher depression scores than those living in Sichuan, Shandong, Shanghai Zhejiang, Jiangsu, Henan, and Liaoning. While after adjusting the individual-level as well as province-level variables, as shown in Figure 1B, the results have a little changed which the respondents living in Guangdong, Chongqing, Hunan were more likely to report higher depression scores compared to those living in Shandong.

### Robustness Analysis

Finally, we adjusted the method to check the robustness of this study. We referenced Radloff (37) suggestion of viewing the 80th percentile point as the cut-off point for depressive symptoms. Some studies have demonstrated this method being applicable (46, 47). Therefore, we used the 80th percentile point of CES-D total score (score of 8) as the cut-off point for depressive symptoms in this study. According to this standard, we detected 3,965 respondents suffering from depression. Then we viewed the outcome variable as a binary variable and conducted the multilevel logistic regression to re-estimate the model. As shown in Supplementary Table 1, the results were not apparent changes, which argues that our finding was robust.

## Discussion

In this study, we combined the characteristics of individual levels and province-level variables by performing multilevel analysis to study the influencing factors of depression. We re-confirmed the association between individual basic demographic characteristics and depression. Then after controlling individual-level variables, we compared depression in different provinces in China and examined the effect of provincial-level factors on depression.

Overall, our findings are consistent with some previous results at the individual level. This study showed that the occurrence of depression was related to age, gender, education, marital status, BMI, rural/urban residence, and household net income. For example, this study confirms that women are more likely to report high levels of depressive symptoms, consistent with the results of many domestic and external studies (48–50). It was mainly due to biological, psychological, and social status differences between men and women (48, 51). Respondents aged 30–59 years old reported higher depression scores than those aged 16–29 years, while the statistical difference between respondents over 60 years and 16–29 years has become insignificant, which means that the prevalence of depression first increased and then decreased with age increasing. This result was consistent with the results of two studies (52, 53). In China, people aged 30–59 years were at work, facing various sources of pressure, while the people above 60 years were in retirement, which partly explained why people aged 30–59 were more prone to depression. In addition, older people in China tend to have better intergenerational support, which is positively correlated with wellbeing (54). Although a significant statistical difference between respondents over 60 years and 16–29 years has emerged in our robustness check, the participants aged 30–59 years had higher estimated values suggesting they were more likely to suffer from depression compared with aged 16–29 years. This result was not contradictory to the previous analysis.

Divorced or widowed as adverse life events were positively correlated with depression (52), suggesting divorced or widowed as potential risk factors for depression, which was why respondents with divorced or widowed were more prone to depression. Education and income as two indicators of socioeconomic status showed significant association with depression (15). High economic levels and increased education attainment lead to better health conditions and high life satisfaction (55). In this study, respondents with higher education attainment and house income tend to report low depression scores. In China, medical standards, living facilities, and economic conditions in rural are inferior to those in cities, so the health status of rural residents is often not as good as that of urban residents.

Interestingly, we found an inconsistent result with most studies that depression seemed to be significantly negatively associated with BMI. This study demonstrated that the respondents with lower BMI reported higher depression scores. Although many studies suggested that overweight or obesity is a risk factor for depression (56–58), our study results were consistent with one study based on the population of China (59). The studies based on European and American sample populations all reported a positive relationship between BMI and depression, but a few studies were reported in China. Therefore, we boldly suspected it is a cultural factor causing a negative relationship between depression and BMI in China. Just as there is a saying in China that being able to eat is a blessing, obese people are considered to be blessed in China, but it needs further scientific research and proof. At the same time, this also provided an idea for further study of Chinese BMI and depression.

Although factors related to depression have been widely studied, few studies focused on the effects of province factors on depression, especially in China. A total of 34 provincial administrative regions in China. Different provinces often have different levels of economic development, demographic characteristics, and even customs, making participants between these provinces tend to hold the other features endowed by province-level provinces. This means participants in the same province tend to be more similar, while the variation of participants from different provinces often is larger. So, general linear or logistic regression will not be appropriate for this situation (41). We examined the effects of province factors in China on depression in this study utilizing the multilevel statistical analysis. There has been considerable macro-economic evidence that national economic figures relate systematically to the nation's health (60). Our study found that the respondents living in provinces with lower rural and urban household income inequality and higher people's wellbeing reported lower depression scores.

The income gap between urban and rural residents has always existed because of China's unique dual household registration system (40) and other related factors; the income gap between urban and rural residents has always existed. However, few studies paid attention to the association between the income gap between urban and rural residents and overall depression. Many studies suggest that income inequality at the province-level was positively related to depression (23, 61). This study also found the same results based on the gap in per capita disposable income between urban and rural residents, which suggested that the Chinese government should care more about rural areas when formulating policies.

The primary purpose of social security and employment expenditure is to reduce the income and property gap, ensure social equity, and maintain social stability (62). This study showed that respondents living in provinces with higher social security and employment expenditure reported lower depression scores. Provinces with higher social security expenditures are better able to handle the problem of the gap between the rich and the poor and ensure social stability. Related research has shown that social stability has a significant positive impact on mental health (63). This result, combined with the above result, suggested that it is necessary to increase expenditure for social security and employment in rural areas.

From Model 4 to Model 6 in Table 2, we found that the effect of GRP per capita on depression was from statistically significant to insignificant, and the estimated values got smaller (β from 0.065 to 0.024). In contrast, the effect of RUR and ESSE on depression kept statistically significant in this process. This could be partly explained by the correlation among three provincial-level factors. But on the other hand, this also reflected essential information that income inequality and people's wellbeing play a more significant role in affecting depression than GRP per capita. This finding is important for related government departments reasonably making policies in the future. They must be cautious that blindly pursuing economic development does not help improve national people's depression. More importantly, they should work to reduce the national income gap and improve the social security expenditures to enhance people's wellbeing.

Through the caterpillar plot, we could observe the overall depression of residents in various provinces. Comparing Figures 1A,B, we found that 95% CI of residual of Shaanxi provinces, Gansu, and Guizhou have stretched across the zero-horizon line in Figure 1B rather than been above the zero-horizon line in Figure 1A. However, the 95% confidence interval of residual of Guangdong and Chongqing has remained above the zero-horizon line. This result indicated that respondents living in Shaanxi, Gansu, and Guizhou are more likely to report higher depression scores could be partly explained by provinces factors of RUR and ESSE. In comparison, province-level factors have not accounted for respondents living in Guangdong and Chongqing being more likely to report higher depression scores. Therefore, future work is needed to understand why the overall depression in Guangdong and Chongqing was higher than in other provinces in China after adjusting the individual-level characteristic and related province-level factors.

The strength of this study lies in the use of multilevel statistical analysis to estimate the effect of the province- and individual-level factors on depression, which makes estimates values more credible compared with using general linear regression or logistic regression. At the same time, we performed the residual analysis. We plotted the caterpillar plot to show how much depression scores the provinces add for individuals, which can guide our subsequent case investigation. For example, we can further study why the overall depression in Guangdong and Chongqing was higher than in other provinces in China after adjusting the individual-level characteristic and related province-level factors. However, our findings should be considered in light of the following limitations. First, due to difficulties obtaining data at the province-level, we only roughly treated the RUR to measure the degree of rural and urban household income inequality, which was more or less arbitrary. Second, the factors that affect depression were complex, while we only included variables mentioned above because of the limitation of data. So, we could not ignore the fact that many unobserved variables existed. Finally, this study was a cross-sectional study, and we could not offer a causal explanation of various variables and depression.

This study re-confirms the association between individual-level characteristics and depression based on the most recent wave of CFPS in 2018. And based on this, province-level factors were introduced to this study. Our findings could have important implications for the government making macro policies to reduce the damage of depression and improve and prevent depression in the population more comprehensively.

## Data Availability Statement

The original contributions presented in the study are included in the article/Supplementary Material, further inquiries can be directed to the corresponding authors.

## Ethics Statement

The studies involving human participants were reviewed and approved by Management Committee of China Social Science Survey Center, Peking University approved the study protocol. Written informed consent to participate in this study was provided by the participants' legal guardian/next of kin.

## Author Contributions

YG, NW, and DL were involved in the study conceptualization and study design. NW and JX carried out statistical analyses. The first draft of the article was written by NW and TY. YL, HY, HW, DL, and TY provided critical revision of the article for important intellectual content. All the authors contributed to the interpretation of the data and approved the final version of the article.

## Funding

This study was supported by the Chengdu High-level Key Clinical Specialty Construction Project.

## Conflict of Interest

The authors declare that the research was conducted in the absence of any commercial or financial relationships that could be construed as a potential conflict of interest.

## Publisher's Note

All claims expressed in this article are solely those of the authors and do not necessarily represent those of their affiliated organizations, or those of the publisher, the editors and the reviewers. Any product that may be evaluated in this article, or claim that may be made by its manufacturer, is not guaranteed or endorsed by the publisher.

## Supplementary Material

Supplementary Table 1 Robustness check: using the CES-D score of 8 as the threshold value of suffering depressive symptoms.
