# Supplementary material for: Province- and Individual-Level Influential Factors of Depression: Multilevel Cross-Provinces Comparison in China
Source: Front Public Health. 2022 May 6;10:893280. doi: 10.3389/fpubh.2022.893280 (PMC9120660; doi:10.3389/fpubh.2022.893280)
Supplement: Supplementary Table 1 — Robustness check: using the CES-D score of 8 as the threshold value of suffering depressive symptoms. [file Table_1.pdf]

Supplementary Table 1. Robustness check: using the CES-D score of 8 as the threshold value of suffering depressive symptoms.

**Variables**	Depressive symptoms (CES-D>8)					
	Model 1	Model 2	Model 3	Model 4	Model 5	Model 6
Individual-level variables						
**Gender (ref: female)**						
male		0.754*** (0.029)	0.754*** (0.029)	0.754*** (0.029)	0.753*** (0.029)	0.752*** (0.029)
**Age group (ref: 16-29)**						
30-44		1.395*** (0.108)	1.398*** (0.108)	1.395*** (0.108)	1.397*** (0.108)	1.400*** (0.109)
45-59		1.526*** (0.122)	1.532*** (0.122)	1.527*** (0.122)	1.533*** (0.122)	1.541*** (0.123)
60-74		1.294** (0.111)	1.303** (0.112)	1.296** (0.111)	1.301** (0.111)	1.312** (0.112)
Above 74		1.167 (0.140)	1.175 (0.141)	1.169 (0.140)	1.179 (0.142)	1.188 (0.143)
**Marital status (ref: married & living with spouse)**						
Unmarried		1.093 (0.086)	1.093 (0.086)	1.094 (0.086)	1.097 (0.086)	1.096 (0.086)
Divorced or widowed		1.791*** (0.125)	1.790*** (0.124)	1.791*** (0.124)	1.793*** (0.125)	1.793*** (0.125)
**Educational attainment (ref: no formal education)**						
Primary school		0.736*** (0.041)	0.738*** (0.041)	0.736*** (0.041)	0.740*** (0.041)	0.742*** (0.041)
Junior high		0.665*** (0.038)	0.668*** (0.038)	0.665*** (0.038)	0.669*** (0.038)	0.672*** (0.038)
Senior high		0.517*** (0.035)	0.519*** (0.035)	0.517*** (0.035)	0.522*** (0.035)	0.525*** (0.036)

College or higher		0.480***	0.483***	0.480***	0.483***	0.487***
		(0.056)	(0.056)	(0.056)	(0.056)	(0.057)
**Rural/Urban residence (ref: rural)**						
Urban		0.900**	0.903*	0.901*	0.907*	0.910*
		(0.036)	(0.037)	(0.037)	(0.037)	(0.037)
**BMI (ref:18.5-23.9)**						
Below 18.5		1.273***	1.272***	1.273***	1.276***	1.273***
		(0.082)	(0.082)	(0.082)	(0.082)	(0.082)
24-27.9		0.855***	0.855***	0.855***	0.855***	0.855***
		(0.037)	(0.037)	(0.037)	(0.037)	(0.037)
28 and above		0.814**	0.814**	0.814**	0.814**	0.814**
		(0.058)	(0.058)	(0.058)	(0.057)	(0.058)
**Per capita household net income (ref: quartile 1)**						
quartile2		0.744***	0.746***	0.745***	0.743***	0.745***
		(0.036)	(0.036)	(0.036)	(0.036)	(0.036)
quartile3		0.614***	0.618***	0.615***	0.613***	0.616***
		(0.033)	(0.033)	(0.033)	(0.033)	(0.033)
quartile4		0.645***	0.651***	0.649***	0.643***	0.646***
		(0.041)	(0.042)	(0.042)	(0.041)	(0.041)
Province-level variables						
RUR			1.433**			1.282*
			(0.176)			(0.135)
GDP				0.987*		1.006
				(0.017)		(0.014)
ESSE					0.995***	0.995***
					(0.001)	(0.001)
Constant	1.074**	1.053**	1.036**	1.051**	1.019*	1.015*
	(0.025)	(0.018)	(0.013)	(0.018)	(0.008)	(0.006)
N	19072	19072	19072	19072	19072	19072

Abbreviation: BMI: Body Mass Index; CES-D8: the eight-item short version of the Center for Epidemiologic Studies Depression Scale; ESSE: Expenditure for Social Security and Employment; GRP: Gross Regional Product; RUR: the Ratio of Per Capita Disposable Income of Urban and Rural.

Notes: Odds ratio and standard errors (standard errors in parentheses); * $P < 0.05$, ** $P < 0.01$, *** $P < 0.001$.
